# Pharmacokinetics and Pharmacokinetic/Pharmacodynamic Integration of Enrofloxacin Following Single Oral Administration of Different Doses in Brown Trout (*Salmo trutta*)

**DOI:** 10.3390/ani11113086

**Published:** 2021-10-28

**Authors:** Kamil Uney, Ertugrul Terzi, Duygu Durna Corum, Rahmi Can Ozdemir, Soner Bilen, Orhan Corum

**Affiliations:** 1 Department of Pharmacology and Toxicology, Faculty of Veterinary Medicine, University of Selcuk, Konya 42031, Turkey; kuney@selcuk.edu.tr; 2 Faculty of Fisheries, University of Kastamonu, Kastamonu 37200, Turkey; ertugrulterzi@gmail.com (E.T.); rozdemir@kastamonu.edu.tr (R.C.O.); sbilen@kastamonu.edu.tr (S.B.); 3 Department of Pharmacology and Toxicology, Faculty of Veterinary Medicine, University of Kastamonu, Kastamonu 37200, Turkey; ddurna@kastamonu.edu.tr

**Keywords:** brown trout, enrofloxacin, pharmacokinetics, pharmacodynamics

## Abstract

**Simple Summary:**

The pharmacokinetic/pharmacodynamic studies report the use of enrofloxacin at higher doses than 10 mg/kg in fish. Pharmacokinetic data for increasing doses of enrofloxacin can facilitate suggestions regarding the dose for the treatment of infections in brown trout. This study aims to determine single oral pharmacokinetics of enrofloxacin at 10, 20, and 40 mg/kg doses in brown trout and pharmacodynamics against *Aeromonas hydrophila* and *A. sobria*. Enrofloxacin exhibited non-linear and dose-disproportional pharmacokinetics. The long action of enrofloxacin following the single oral administration at 10 and 20 mg/kg doses may provide the unique dosage regimen to minimize handling, thereby reducing the cost of administration and stress in brown trout.

**Abstract:**

The pharmacokinetic of enrofloxacin was investigated in brown trout (*Salmo trutta*) following oral administration of 10, 20, and 40 mg/kg doses at 11 ± 1.5 °C. Furthermore, MICs of enrofloxacin against *Aeromonas hydrophila* and *A. sobria* were determined. The plasma concentrations of enrofloxacin and ciprofloxacin were determined using HPLC–UV and analyzed by non-compartmental method. Following oral administration at dose of 10 mg/kg, total clearance (CL/F), area under the concentration–time curve (AUC_0−__∞_) and peak plasma concentrations (C_max_) were 41.32 mL/h/kg, 242.02 h*μg/mL and 4.63 μg/mL, respectively. When compared to 10 mg/kg dose, the dose-normalized AUC_0–__∞_ and C_max_ were increased by 56.30% and 30.08%, respectively, while CL/F decreased by 38.4% at 40 mg/kg dose, suggesting the non-linearity. Ciprofloxacin was not detected in the all of plasma samples. The MIC values of enrofloxacin were ranged 0.0625–4 μg/mL for *A. hydrophila* and 0.0625–2 μg/mL for *A. sobria*. The oral administration of enrofloxacin at 10 (for 192 h) and 20 (for 240 h) mg/kg doses provided the AUC of enrofloxacin equal to 1.23 and 1.96-fold MICs, respectively, for *A. hydrophila* and *A. sobria* with the MIC_90_ values of 1 µg/mL. However, further researches are needed on the PK/PD study of enrofloxacin for the successful treatment of infections caused by *A. hydrophila* and *A. sobria* in brown trout.

## 1. Introduction

Aquaculture has become an important sector in global food production, due to changes in human dietary habits for a healthy diet. Fish is an important part of a healthy diet because of its low content of cholesterol, carbohydrates, and saturated fats, and its high content of proteins and essential nutrients [1,2]. With the increase in fish production in farms, bacterial diseases have become widespread due to factors such as inadequate management and poor environmental conditions and stress [1,3]. Therefore, antibiotics are used as prophylactic and therapeutic in bacterial infections in fish [3].

Brown trout (*Salmo trutta*) is a commercially important fish species in our country, which is preferred by customers for its taste and colour [4]. *Aeromonas* spp. are one of the most common gram-negative bacteria in freshwater habitats and cause severe infections in cultured fish species [5]. *A. hydrophila* and *A. sobria* are the etiological factors of motile Aeromonas septicemia, also known as tail and fin rot, in freshwater fish species including trout [6]. Clinical signs of motile Aeromonas septicemia include anemia, hemorrhaging, ulcer, abdominal distension and accumulation of fluid, resulting in mass mortality [6].

Enrofloxacin is a fluoroquinolone group antibacterial drug developed for use in veterinary medicine. It has a broad spectrum of antibacterial activity, including Gram-negative and Gram-positive bacteria, as well as *Mycoplasma* spp. [7,8]. Enrofloxacin has a bactericidal effect by inhibiting the activity of bacterial DNA gyrase (topoisomerase II) and topoisomerase IV enzymes [8]. In both mammalian and nonmammalian species, enrofloxacin is dealkylated to ciprofloxacin, which exhibits a potency and spectrum of activity similar to enrofloxacin [7]. Enrofloxacin is widely used in fish due to its properties such as good absorption, large volume of distribution, high bioavailability, and long terminal half-life [9,10]. Although enrofloxacin is included in category B (Restrict) in the classification of antibiotics for veterinary use in terms of the risk of antimicrobial resistance by the EMA, it has been stated that it can be used in fish in cases in which there are few alternative treatment options, such as Aeromonas infection [11]. Enrofloxacin is used in diseases such as septicaemia, furunculosis, enteric red mouth, vibriosis or skin diseases in fish due to its effectiveness against bacteria such as *Aeromonas* spp., *Vibrio* spp., and *Yersinia* spp. [12,13].

The antibacterial effect of enrofloxacin is concentration-dependent, and the pharmacokinetic (PK) and pharmacodynamic (PD) index is used to determine its antimicrobial activity [14]. The most commonly used PK-PD indices for enrofloxacin are the area under the concentration-versus time curve (AUC)/minimum inhibitory concentration (MIC) and peak plasma concentration (C_max_)/MIC and the desired level of these indices is important for clinical success and prevention of antimicrobial resistance development [15]. Although the PK-PD relationship of enrofloxacin was determined for *A. hydrophila* in carp [16], no studies were found in brown trout. The traditional dose of enrofloxacin in fish is 10 mg/kg [17]. In fish, the PK/PD studies recommended the use of enrofloxacin at 10 mg/kg dose for bacteria MIC of ≤0.40 µg/mL [18]. However, it has been reported that higher doses of enrofloxacin need for bacteria with MIC of 0.41 µg/mL to 2 μg/mL with the resistance breakpoint [16]. Therefore, the determination of enrofloxacin PKs and PD for increasing doses will be necessary to facilitate suggestions regarding the dose of enrofloxacin for the treatment of infections caused by susceptible pathogens in brown trout. The aims of this study were (I) to determine the PK profile of enrofloxacin in brown trout following oral administration at the doses of 10, 20 and 40 mg/kg at 11 ± 1.5 °C; (II) to determine the MICs of enrofloxacin against *A. hydrophila* and *A. sobria* isolated from fish; and (III) to integrate PK/PD data in order to predict enrofloxacin efficacy.

## 2. Materials and Methods

### 2.1. Chemicals

The analytical standard of enrofloxacin (≥99%) and ciprofloxacin (≥98%) were purchased from Sigma-Aldrich (St. Louis, MO, USA). Acetonitrile, triethylamine, and orthophosphoric acid were supplied by Merck (Darmstadt, Germany). The ultra-pure water was obtained through a distilled water system (Aqua Maxi-Ultra System; Younglin Instrument Co., Ltd., Hogye-dong, Anyang, Korea).

### 2.2. Animals

Three hundred and six healthy brown trout (*Salmo trutta*), originated from the Eastern Black Sea Region, weighing 70–80 g were supplied from a local fish farm (Kastamonu, Turkey). Fish were examined to be clinically healthy based on body condition and behavior, with no visible signs of trauma, or illness before inclusion in the study. Fish were distributed randomly into 12 concrete tanks (500 L) in the fish farm, and each tank with a continuous flow through spring water contained maximum twenty-six fish. The fish were kept under normal daily lighting conditions (daylight and dark) with a pH of 8.0 ± 0.3 and water temperature of 11 ± 1.5 °C. They were fed a drug-free commercial diet (Sibal Yem, Sinop, Turkey) every day, and fish were not fed for 12 h before and after drug administration. The fish were acclimated for 15 days prior to the study. The experiment was approved by the Kastamonu University Animal Experiments Local Ethics Committee, Turkey, and carried out in accordance with the European Directive (2010/63/UE).

### 2.3. Experimental Design

Fish were randomly divided into three groups according to the 10, 20 and 40 mg/kg doses of enrofloxacin. The formulation (Baytril 10%, Oral solution, Bayer, Istanbul, Turkey) of enrofloxacin was diluted to a concentration of 5 mg/mL using sterile water for oral administration. Enrofloxacin solution was administered by oral gavage at 10 (n = 102), 20 (n = 102), and 40 mg/kg (n = 102) doses. Blood samples (about 0.6–0.8 mL) were taken from the caudal vein of six fish by use of a 26-G needle attached to 1 mL syringe and placed into heparin tubes at the following times: 0, 0.25, 0.5, 1, 2, 4, 8, 12, 24, 48, 72, 96, 120, 144, 168, 192, and 240 h after oral administration. The drug administration and blood samples were performed after the fish were anesthetized in water containing 200 mg/L tricaine methane sulfonate (MS-222). Blood samples were centrifuged at 4000× *g* for 10 min, and the plasma obtained was stored at −80 °C until analysis. All plasma samples were analyzed for enrofloxacin content within 2 months after treatments concluded.

### 2.4. HPLC and Chromatographic Conditions

**Figure 1:**
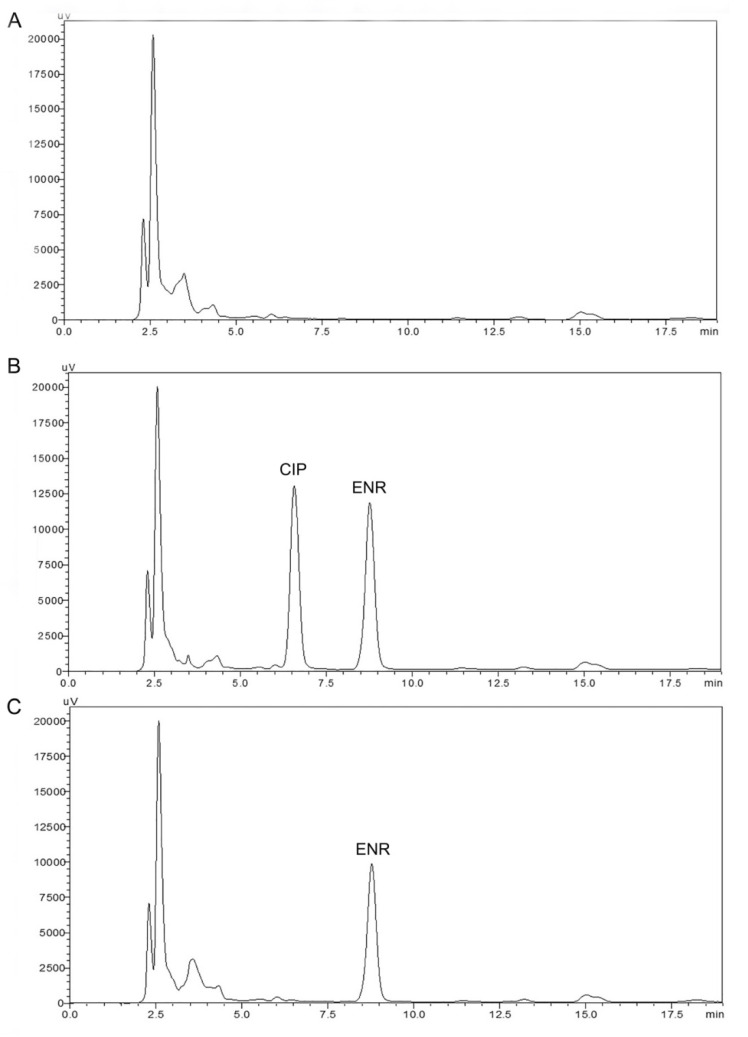
Representative chromatograms of blank brown trout plasma (**A**); brown trout plasma spiked with enrofloxacin and ciprofloxacin (10 µg/mL) (**B**); and brown trout plasma sample at 8 h after oral administration of enrofloxacin at dose of 20 mg/kg (**C**). ENR, enrofloxacin, CIP, ciprofloxacin.

The plasma concentrations of enrofloxacin and ciprofloxacin were assayed according to the previously reported high-performance liquid chromatography–ultraviolet detection (HPLC–UV) method [19]. Briefly, the 200 μL of acetonitrile was added to a 100 μL of plasma sample followed vortexed for 45 s and centrifuged at 10,000× *g* for 12 min. After centrifugation, 100 μL of supernatant was pipetted into a micro-tube and 100 μL of water was added and mixed for 15 s. A total of 200 μL of clear supernatant was transferred to autosampler vials, and 20 μL of was injected into the HPLC system (Shimadzu, Tokyo, Japan). HPLC system consists of a degasser (DGU-20A), a pump (LC-20AT controlled by the CBM-20A), an auto-sampler (SIL 20A), and a column oven (CTO-10A). For the detection of enrofloxacin and ciprofloxacin, an SPD-20A UV-VIS detector set to 280 nm wavelength and a Gemini^TM^ C18 column (250 × 4.6 mm; internal diameter, 5 μm; Phenomenex, Torrance, USA) were used. The column and autosampler temperatures were maintained at 40 °C and 24 °C, respectively. The mobile phase with a flow rate of 1 mL/min consisted of acetonitrile (18%) and 0.4% orthophosphoric acid containing 0.4% triethylamine (82%). Data analysis was performed with the LC solution software program (Shimadzu, Tokyo, Japan). Enrofloxacin and ciprofloxacin eluted at approximately 6.5 and 8.5 minutes, respectively, with a total run time of 19 minutes (Figure 1).

The chromatographic method was validated according to EMA (2011) guidelines [20]. The stock solution of enrofloxacin and ciprofloxacin was prepared in 0.01 M NaOH to obtain a concentration of 1 mg/mL. Calibration standards (0.04–40 μg/mL) and quality control samples were prepared by adding standard solutions of enrofloxacin and ciprofloxacin into blank fish plasma. The calibration standards of enrofloxacin and ciprofloxacin prepared from blank fish plasma at a concentration between 0.04 and 40 μg/mL were linear (R2 > 0.9992). The quality control samples, which were prepared in six replicate analyses of each level at the concentration of 0.1, 1 and 10 μg/mL within 1 day or on 6 consecutive days, were used to determine the recovery, precision, and accuracy. The recoveries of enrofloxacin and ciprofloxacin ranged from 91% to 104%. The lower limit of quantification was 0.04 µg/mL for enrofloxacin and ciprofloxacin in brown trout plasma with the coefficient of variation less than 20% and the bias of ±15%. The intraday and interday coefficients of variation for enrofloxacin and ciprofloxacin were ≤6.7% and ≤7.8%, respectively. The biases calculated for intraday and interday accuracy for enrofloxacin and ciprofloxacin ranged from −7.2 to 6.4%.

### 2.5. Pharmacokinetic Analysis

The plasma concentration–time curves of enrofloxacin was plotted using the WinNonlin 6.1.0.173 software (Pharsight Corporation, Scientic Consulting Inc., Sunnyvale, CA, USA). Pharmacokinetic parameters of enrofloxacin were determined by the non-compartmental analysis using mean plasma concentration values obtained after administrations of 10, 20 and 40 mg/kg doses. The terminal elimination half-life (t_1/2λz_), mean residence time (MRT), area under the plasma concentration–time curve (AUC), AUC extrapolated from t_last_ to ∞ in % of the total AUC (AUC_extrap_ %)_,_ total clearance (CL/F) and apparent volume of distribution (V_darea_/F) were calculated. The peak plasma concentration (C_max_) and the time to reach C_max_ (T_max_) were determined from observed data on the plasma concentration–time curve.

### 2.6. Determination of MICs

Broth microdilution method of Clinical and Laboratory Standards Institute [21] was used to determine the MICs of enrofloxacin for fish pathogenic bacteria *(A. hydrophila* and *A. sobria*) isolated from fish farm. For this purpose, 9 different concentrations of enrofloxacin (from 8 to 0.031 μg/mL) were prepared in Mueller Hinton Broth, and a total of 150 μL of each concentration per well was put into the 96-well plate and used for the MIC test at 12 °C. The lowest concentration of enrofloxacin that visibly inhibited bacterial growth was accepted as MIC. The MIC_50_ and MIC_90_ represent the MIC value at which at least 50% and 90% of the isolates, respectively, are inhibited. The MIC_50_ and MIC_90_ of enrofloxacin were calculated using “n × 0.5” and “n × 0.9”, respectively, in which “n” was the number of test strains [22].

### 2.7. PK/PD Integration

The AUC_0–last_/MIC_90_ was determined using the MIC_90_ values of enrofloxacin for *A. hydrophila* and *A. sobria* and the PK parameters following oral administration of enrofloxacin at a dose of 10 and 20 mg/kg.

### 2.8. Statistical Analysis

Plasma concentrations were presented as mean ± standard deviation values. This study presents the PK parameters of enrofloxacin that were calculated using mean plasma concentration values obtained after drug administration of 10, 20 and 40 mg/kg doses [23]. The following formula was used to evaluate the differences in the PK parameters based on the different doses: (100 × (Value obtained following X dose−Value obtained following Y dose)/Value obtained following Y dose). The values of ≤%(−)25 and ≥%(+)25 values were accepted as significant [24,25].

## 3. Results

**Figure 2:**
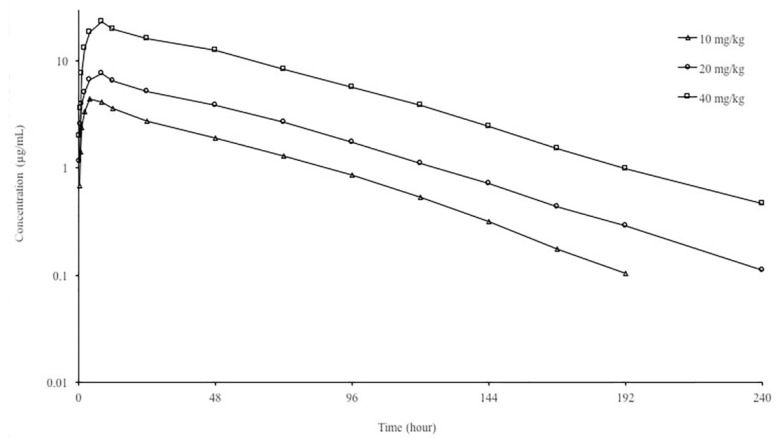
Semi-logarithmic plasma concentration-time curves of enrofloxacin following single oral administration at doses of 10, 20, and 40 mg/kg in brown trout at 11 ± 1.5 °C (mean, n = 6).

In this study, enrofloxacin oral administered at a dose of 10, 20 and 40 mg/kg in brown trout was well tolerated and had no serious adverse effect. Semi-logarithmic plasma concentration-versus-time curves and PK parameters of enrofloxacin following oral administrations of 10, 20 and 40 mg/kg dose in brown trout are presented in Figure 2 and Table 1, respectively. Enrofloxacin was measured up to 192 h for 10 mg/kg dose and 240 h for 20 and 40 mg/kg doses in plasma. Following oral administration at dose of 10 mg/kg, t_1/2ʎz_, CL/F, V_darea_/F, AUC_0−__∞_ and C_max_ were 33.82 h, 41.32 mL/h/kg, 2.02 L/kg, 242.02 h*μg/mL and 4.63 μg/mL, respectively. There was no difference in pharmacokinetic parameters at 20 mg/kg compared to 10 mg/kg. When compared to 10 mg/kg dose, the dose-normalized AUC_0–∞_ and C_max_ were increased by 56.30% and 30.08% respectively, while CL/F decreased by 38.4% at 40 mg/kg dose, suggesting the non-linearity. Ciprofloxacin was not detected in the plasma of fish in all dose groups.

The MIC values of enrofloxacin for *A. hydrophila* and *A. sobria* isolated from fish were presented in Table 2. The MIC values of enrofloxacin were 0.0625–4 μg/mL for A*. hydrophila* and 0.0625–2 μg/mL for *A. sobria*. For *A. hydrophila* and *A. sobria* isolated from the fish farm, the MIC_50_ value was 0.25 and 0.5 µg/mL, respectively, and the MIC_90_ value was 1 µg/mL. For 10 and 20 mg/kg doses, the the AUC_0-last_/MIC_90_ estimated using the in vitro MIC_90_ data (1 µg/mL) for *A. hydrophila* and *A. sobria* and the in vivo PK parameters of enrofloxacin was 236.92 h and 469.49 h, respectively.

## 4. Discussion

In this study, the PK values obtained after increasing doses of enrofloxacin may contribute to the selection and dosage of enrofloxacin in brown trout. However, this study has some significant limitations that may affect its evaluation. Brown trout received at the single dose of enrofloxacin. However, the number of doses administered must be noticed as it would be necessary to perform in the future a pharmacokinetic and PK/PD integration after multiple administrations according to the indications for use of enrofloxacin in fish. Enrofloxacin was administered via single oral gavage to determine the exact dose and to prevent possible drug loss due to feed in brown trout. The most preferred method of administration is medicated feed due to the ease of application and low labor force in fish [26,27]. Although our preferred method of oral gavage is impractical for large numbers of fish, it can be used therapeutically in sick animals. In this study, the PK of enrofloxacin was performed on healthy brown trout. However, the PKs of enrofloxacin showed significant differences in crucian carp infected with *A. hydrophila* [16] and in broilers infected with *Escherichia coli* [15]. The PK study was performed with brown trout reared at 11 ± 1.5 °C. Because the water temperature affects the PKs of enrofloxacin in fish [9], the results obtained in this study may differ for brown trout grown at different water temperatures. To minimize trauma and stress in brown trout, blood collection and drug administration were carried out under anesthesia by experienced personnel. However, the anesthesia may have altered the PK of enrofloxacin by reducing the stress response [28]. However, the PK of florfenicol has not been alter significantly in Nile tilapia exposed to MS-222 of 300 mg/L [29]. 

The traditional dose of enrofloxacin in fish is 10 mg/kg, and it has been used at doses between 5 and 80 mg/kg in previous studies [17,30,31]. After a single oral administration of enrofloxacin at different doses (10, 20 and 40 mg/kg) in rainbow trout, no biochemical and histopathological changes were observed, except for epithelial hyperplasia in the gill at a dose of 40 mg/kg [32]. It has been reported that enrofloxacin is well tolerated in the crucian carp and Siberian sturgeon at a dose of 40 mg/kg, but it can cause toxicity above this dose [16,31]. Therefore, oral doses of 10, 20 and 40 mg/kg of enrofloxacin were selected in brown trout and no adverse effects were observed.

The t_1/2ʎz_ of enrofloxacin following oral administration to brown trout ranged from 33.82 h (10 mg/kg dose) to 40.68 h (40 mg/kg dose) at 11 ± 1.5 °C, which was similar to that previously reported for rainbow trout (42.98 h, 16.3 °C) [14], grass carp (42.72 h, 22 °C) [33] and snakehead fish (35.80 h, 24–26 °C) [34], and shorter than that reported in rainbow trout (78.8 h, 15 °C) [35] and crucian carp (67.69 h, 28 °C) [16]. In addition, it was longer than that reported in brown trout (22.09 h, 10 °C) [4]. The reasons for this difference in the same fish strain may be variations between fish size (70–80 g vs. 170 ± 32 g), blood sampling times (240 h vs. 120 h) and the sensitivity of analytical method (0.04 µg/mL vs. 0.1 µg/mL). The t_1/2ʎz_ following the administration of 20 and 40 mg/kg doses in brown trout was unimportantly prolonged by 10.32 and 20.28%, respectively, according to 10 mg/kg. However, after the administration of enrofloxacin in increasing doses (10, 20 and 40 mg/kg) in grass carp, t_1/2ʎz_ increased from 42.72 h to 97.36 h [33].

Enrofloxacin showed wide V_darea_/F ranging from 1.55 to 2.26 L/kg following administration at doses of 10, 20, and 40 mg/kg. Enrofloxacin has a large V_d_ of 2.56 to 5.75 L/kg in fish due to its high lipophilic solubility and low (≤8.8%) plasma protein binding [16,17,30]. In this study, according to 10 mg/kg dose, the V_darea_/F of enrofloxacin in brown trout unimportantly increased by 11.88% at 20 mg/kg dose and decreased by 23.27% at 40 mg/kg dose. In similar, compared to 20 mg/kg dose, the V_darea_ of enrofloxacin in liver and kidney of Nile tilapia was decreased by 21.8–23% at the 40 mg/kg dose [36]. However, it noticeably decreased by 52.8–61.8% at the 80 mg/kg dose [36]. These data indicate that the V_darea_/F of enrofloxacin may change with increasing dose.

In this study, according to 10 mg/kg dose, the CL/F of enrofloxacin increased insignificantly by 1.79% at 20 mg/kg while decreased by 38.43% at 40 mg/kg. In Nile tilapia, the liver and kidney CL/F of enrofloxacin at 40 and 80 mg/kg doses noticeably decreased by 37.29–53.75% and 76.72–87.21%, respectively according to 20 mg/kg dose [36]. In addition, as the dose of enrofloxacin increased, plasma CL/F did not change in crucian carp, while grass carp decreased [16,33]. Enrofloxacin is converted to ciprofloxacin by oxidative deethylated by the P450 system in mammals [8]. In aquatic animals, the conversion of enrofloxacin to ciprofloxacin is low compared with terrestrial animals [37]. In our study, ciprofloxacin could not be detected in plasma samples taken after enrofloxacin administration. Although ciprofloxacin was detected at a low ratio in largemouth bass [10], crucian carp [38], grass carp [33] and rainbow trout [35], it was not detected in brown trout [4], rainbow trout [14] and sea bream [38]. It has been stated that the single dose administration of enrofloxacin in sea bass strongly inhibits the hepatic P450 enzymes system [37]. In addition, differences such as fast, gender, sexual maturity and size in fish may affect biotransformation activity [39]. The above-mentioned reasons may explain the difference in detection of ciprofloxacin in fish species. Enrofloxacin is excreted primarily by the kidney by glomerular filtration and tubular secretion. It has been reported that the PKs of enrofloxacin are unchanged in hepatic impairment, but significantly altered in renal failure [40]. Organic anions and organic cation transports play a role in the renal tubular secretion of enrofloxacin [41]. The active transport system of organic anions and cation is mediated by carrier proteins, and this transport mechanism can be saturated [42]. In this study, the CL/F of enrofloxacin may have decreased due to the enzyme inhibition and the saturation of the active transport system at a dose of 40 mg/kg.

Following oral administration of enrofloxacin at a dose of 10 mg/kg in brown trout, C_max_ and AUC_0-__∞_ were 4.63 µg/mL and 242.02 h*µg/mL, respectively. After oral administration of enrofloxacin at a dose of 10 mg/kg to different fish species, C_max_ and AUC_0-__∞_ ranged between 0.55–5.2 µg/mL and 39.88–271.6 h*µg/mL, respectively [30,35]. Compared to the 10 mg/kg dose, the dose normalized C_max_ and AUC_0-__∞_ decreased unimportantly by 15.12% and 1.77%, respectively, at the 20 mg/kg dose, and increased significantly by 30.08% and 56.30%, respectively, at the 40 mg/kg dose. The AUC_0–∞_ increased disproportionally large at the 40 mg/kg compared to those of 10 and 20 mg/kg. In other words, the dose-normalized AUC_0–∞_ (AUC/dose) of the 10, 20, and 40 mg/kg, which were 24.2, 23.8, and 37.8 h*µg/mL, respectively, strongly indicated the existence of non-linear PK at the highest dose (40 mg/kg). Moreover, the increased t_1/2ʎz_ and the decreased CL/F at the 40 mg/kg compared to the two lower doses also supported this conclusion because the t_1/2ʎz_ and CL/F should be constant (dose-independent) in the case of linear PK (which is true for the 10–20 mg/kg). Finally, the increase in the AUC/dose and t_1/2ʎz_ and the decrease in CL/F implied the saturable drug elimination as the mechanism behind the observed non-linear PK. In rats, enrofloxacin at higher doses (150 mg/kg) showed non-linear PKs after subcutaneous administration [43]. However, after oral administration of enrofloxacin at doses of 5–40 mg/kg in crucian carp, the C_max_ and AUC was found to be linear [16]. Dose linearity and proportionality are important in estimating the effects of dose adjustments [16]. Therefore, the linear PKs of enrofloxacin at doses of 10 and 20 mg/kg is important in clinical use in brown trout. In this study, the T_max_ of enrofloxacin was 5.04 h after oral administration of 10 mg/kg at 11 ± 1.5 °C. T_max_ of enrofloxacin after oral administration at 10–28 °C in different fish species varied between 0.5 and 49.2 h [4,16,35]. The variation of T_max_ in fish species may be due to differences in anatomical, physiological and water temperature [12]. In this study, the T_max_ of enrofloxacin in brown trout was not significantly different at 10, 20, and 40 mg/kg doses.

In this study, MIC values of enrofloxacin were 0.063–4 μg/mL for *A. hydrophila* and 0.063–2 μg/mL for *A. sobria*. The MIC value of enrofloxacin was reported as 0.5 μg/mL for *A. sobria*, and *A. hydrophila* isolated from carp [16,33,44]. In the samples taken from rainbow trout and environmental strains, the MIC value of enrofloxacin for Aeromonas spp., were found to be 0.008–4 μg/mL [44,45]. In this study, for *A. hydrophila* and *A. sobria* isolated from the fish farm, the MIC_50_ value was 0.25 and 0.5 µg/mL, respectively, and the MIC_90_ values were calculated as 1 µg/mL for both species. Enrofloxacin was measured up to 192 h for 10 mg/kg dose and 240 h for 20 mg/kg dose in plasma. The AUC_0-last_ for 10 and 20 mg/kg doses was calculated from time 0 to the last measurable concentration. These data showed a long action of enrofloxacin following the single oral administration in brown trout. The AUC_0–24_/MIC for long-action antibiotics following single dose administration cannot be directly compared with those derived from human medicine, which are almost invariably for 24 h [46]. An alternative and preferred approach is to determine the scaling factor, by dividing the AUC_0-last_/MIC by the time of last measurable concentration [46]. Because the AUC_0–∞_ increased disproportionally large at the 40 mg/kg, the AUC_0-last_/MIC_90_ of enrofloxacin was not calculated. For 10 and 20 mg/kg doses, the AUC_0-last_/MIC_90_ of enrofloxacin was 236.92 h and 469.49 h, respectively, for *A. hydrophila* and *A. sobria* with the MIC_90_ values of 1 µg/mL. The scalars for 10 and 20 mg/kg doses was 1.23 and 1.96, respectively, for *A. hydrophila* and *A. sobria*. The scalars indicate that the AUC of enrofloxacin at 10 (for 192 h) and 20 (for 240 h) mg/kg doses was equal to 1.23 and 1.96-fold MICs, respectively, for *A. hydrophila* and *A. sobria*. Because the ideal scaling factor of enrofloxacin for *A. hydrophila* and *A. sobria* is not known, the results of the present study could not be evaluated. Therefore, the PK/PD study of enrofloxacin is needed to determine the ideal scaling factor for the successful treatment of infections caused by *A. hydrophila* and *A. sobria*.

## 5. Conclusions

The study revealed the dose-dependent PK of enrofloxacin for 10, 20 and 40 mg/kg doses in brown trout reared at 11 ± 1.5 °C. The oral administration of 40 mg/kg resulted in a larger AUC/dose, longer t_1/2ʎz_, and slower CL/F, suggesting the non-linearity. The long action of enrofloxacin following the single oral administration at 10 and 20 mg/kg doses may provide the unique dosage regimen to minimize handling, thereby reducing the cost of administration and stress in brown trout. The oral administration of enrofloxacin at 10 (for 192 h) and 20 (for 240 h) mg/kg doses provided the AUC of enrofloxacin equal to 1.23 and 1.96-fold MICs, respectively, for *A. hydrophila* and *A. sobria* with the MIC_90_ values of 1 µg/mL. However, further researches are needed on the PK/PD study of enrofloxacin for the successful treatment of infections caused by *A. hydrophila* and *A. sobria* in brown trout. 

## Figures and Tables

**Table 1:** Plasma pharmacokinetic parameters of enrofloxacin following single oral administration at doses of 10, 20, and 40 mg/kg in brown trout at 11 ± 1.5 °C (n = 6).

Parameters	10 mg/kg	20 mg/kg	40 mg/kg
t_1/2ʎz_ (h)	33.82	37.31	40.68
AUC_0–24_ (h*µg/mL)	82.02	145.07	433.27
AUC_0–last_ (h*µg/mL)	236.92	469.49	1485.59
AUC_0–∞_ (h*µg/mL)	242.02	475.50	1513.09
AUC_extrap_ (%)	2.11	1.26	1.82
MRT_0–∞_ (h)	54.03	59.47	63.23
CL/F (mL/h/kg)	41.32	42.06	25.44
V_darea_/F (L/kg)	2.02	2.26	1.55
C_max_ (µg/mL)	4.63	7.86	24.09
T_max_ (h)	5.04	6.35	7.13

t_1/2ʎz_, terminal elimination half-life; AUC, area under the plasma concentration–time curve; AUC_extrap_ %, area under the plasma concentration-time curve extrapolated from t_last_ to ∞ in % of the total AUC; MRT, mean residence time; CL/F, total clearance; V_darea_/F, apparent volume of distribution; C_max_, peak plasma concentration, T_max_, time to reach C_max_.

**Table 2:** MIC values of enrofloxacin for *A. hydrophila* and *A. sobria* isolated from fish farm.

Distribution (Numbers of Isolates) of Enrofloxacin MIC (μg/mL)										
Bacterial Species	N	0.031	0.063	0.125	0.25	0.50	1	2	4	8
*A. hydrophila*	13	-	1	1	7	2	1	-	1	-
*A. sobria*	13	-	2	3	1	4	2	1	-	-

MIC; minimum inhibitory concentration, *A. hydrophila*: *Aeromonas hydrophila*, *A. sobria*; *Aeromonas sobria*.

## Data Availability

The data presented in this study are available on request from the corresponding author.
